# Multiscalar cellular automaton simulates in-vivo tumour-stroma patterns calibrated from in-vitro assay data

**DOI:** 10.1186/s12911-017-0461-1

**Published:** 2017-05-30

**Authors:** J. A. Delgado-SanMartin, J. I. Hare, E. J. Davies, J. W. T. Yates

**Affiliations:** 1 Modelling and Simulation, Oncology IMED DMPK, AstraZeneca, Li Ka Shing Centre, Robinson Way, Cambridge, CB2 0RE UK; 2 Bioscience, Oncology IMED, AstraZeneca, Li Ka Shing Centre, Robinson Way, Cambridge, CB2 0RE UK; 3 Physics Department, University of Aberdeen, Aberdeen, UK; 4 GSK R&D Centre, Gunnels Wood Road, Stevenage, SG1 2NY UK

**Keywords:** Cancer, Stroma, Cellular automaton, Oxygen, Hypoxia, Immunohistochemistry, Cell line

## Abstract

**Background:**

The tumour stroma -or tumour microenvironment- is an important constituent of solid cancers and it is thought to be one of the main obstacles to quantitative translation of drug activity between the preclinical and clinical phases of drug development. The tumour-stroma relationship has been described as being both pro- and antitumour in multiple studies. However, the causality of this complex biological relationship between the tumour and stroma has not yet been explored in a quantitative manner in complex tumour morphologies.

**Methods:**

To understand how these stromal and microenvironmental factors contribute to tumour physiology and how oxygen distributes within them, we have developed a lattice-based multiscalar cellular automaton model. This model uses principles of cytokine and oxygen diffusion as well as cell motility and plasticity to describe tumour-stroma landscapes. Furthermore, to calibrate the model, we propose an innovative modelling platform to extract model parameters from multiple in-vitro assays. This platform provides a novel way to extract meta-data that can be used to complement in-vivo studies and can be further applied in other contexts.

**Results:**

Here we show the necessity of the tumour-stroma opposing relationship for the model simulations to successfully describe the in-vivo stromal patterns of the human lung cancer cell lines Calu3 and Calu6, as models of clinical and preclinical tumour-stromal topologies. This is especially relevant to drugs that target the tumour microenvironment, such as antiangiogenics, compounds targeting the hedgehog pathway or immune checkpoint inhibitors, and is potentially a key platform to understand the mechanistic drivers for these drugs.

**Conclusion:**

The tumour-stroma automaton model presented here enables the interpretation of complex in-vitro data and uses it to parametrise a model for in-vivo tumour-stromal relationships.

**Electronic supplementary material:**

The online version of this article (doi:10.1186/s12911-017-0461-1) contains supplementary material, which is available to authorized users.

## Background

Tumours function as complex, interactive, multi-cellular aberrant organs. Within the tumour microenvironment, malignant cells exist amongst extracellular matrix, immune cells, vasculature, lymphatic vessels, fibroblasts, and other stromal cells. This tumour microenvironment can exert pro- and/or anti-tumorigenic actions, depending on context, while malignant cells create a permissive and supportive tumour matrix by secreting stroma-modulating growth factors, including PDGF, EGFR, VEGF, and TGF-β [1]. These factors can activate the surrounding stroma, causing the secretion of additional soluble molecules that mediate extensive cross-talk between the tumour cells and stromal components [2].

Stromal composition, architecture, and quantity varies between patient tumour types, while tumour heterogeneity is a hallmark of patient tumours. It is now well-recognised that complex tumour-stromal interactions underlie tumour growth, progression, and invasion. Recently, it has been demonstrated that the tumour microenvironment can affect tumour pathophysiology [3], therapeutic sensitivity, and response [4, 5]. The recognition of the importance of the stromal compartment in treatment outcome has led to the development of high throughput screens that incorporate a stromal component in addition to a tumour cell component for finding novel therapies. As such, it is necessary to develop further insight into tumour-stroma interactions to aid in the modelling of pre-clinical drug-response data in patient tumours [6–8]. Furthermore, tumour stromal morphology at tissue level was identified as a potential driver of drug efficacy in patient-derived xenografts [9], also identified as determinant of tumour grade in prostate cancers [10]. The origination of these patterns (described in [11]) is in great measure the motivation of this work.

**Fig. 1:**
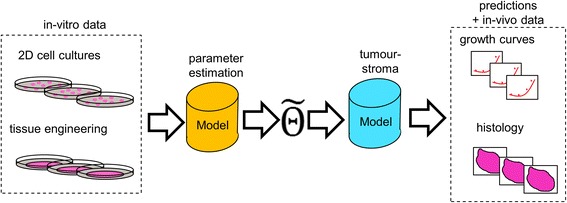
Schema of the models proposed and their integration with data

Despite the important role the tumour microenvironment, few pre-clinical tumour models include an extensive desmoplastic stroma or three dimensional interaction [12]. Here is when simulation work becomes a key translational tool. Recent work in the field of modelling has explored the interactions between tumour-immune cells and nutrients in a multiscalar manner [13–15], also incorporating an in-depth study of intracellular mechanisms [16] and oxygen [17], as well as the role of peripheral anatomical features in disease progression, such as ductal niches [18]. However, to our knowledge, there has been no attempt to predict in-vivo tumour-stroma growth and progression using merely in-vitro assays (see Fig. 1), we consider this a step forward in translational science. For simplicity, we describe two models: 1) the parameter estimation model (PEM) is used to calibrate 2) the tumour-stroma model (TSM), which is a cellular automaton.

## Methods

### Tumour-stroma model (TSM)

**Fig. 2:**
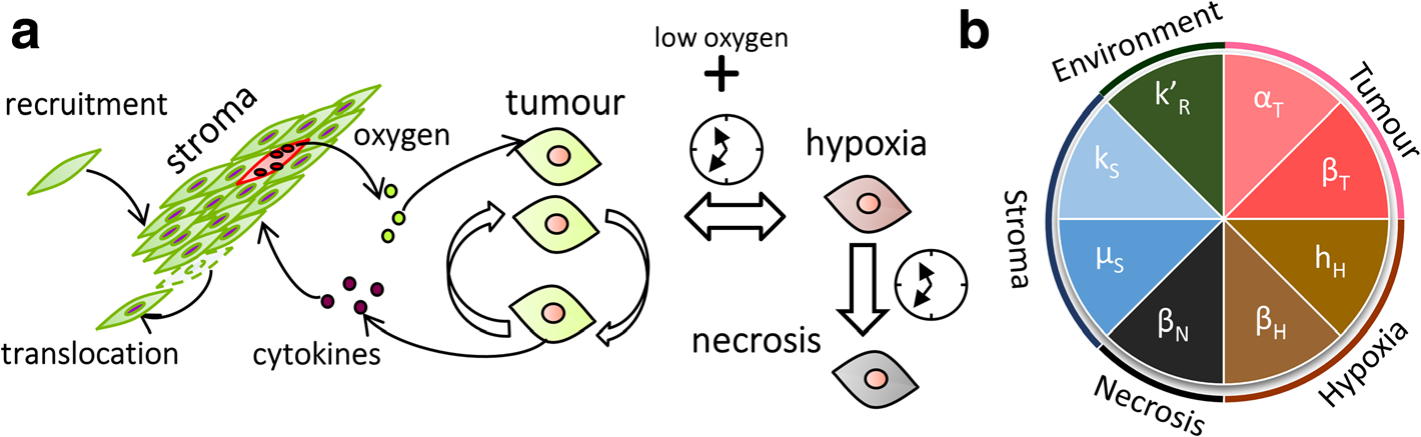
Schema of the TSM showing the valid decisions (**a**) and parameters of the model (**b**)

The TSM is a predictive multiscale lattice-based cellular automaton model described by eight parameters (Figs. 1 and 2b) whose values can be estimated with the parameter estimation model (PEM) defined below. The TSM was inspired by a previously developed oxygen-centred model [19, 20] and the subsequent discovery that tumour-stroma morphology affects therapeutic response [9]. The model considers two cell types: stroma and tumour, whereby the latter can exist in three states: viable, hypoxic, or necrotic. These states depend on the cells’ access to oxygen and a time component (Fig. 2a).

The model is built on a fixed 2D regular lattice in which each square element (or voxel) represents groups in the range of thousands of cells of one kind. These cells, based on their position in space, behave metabolically different. Further, the model considers delays inherent to the biological processes, such as cell cycle progression, which are orchestrated by an internal clock defined by the variable tau (**τ**) in the time domain.

Our virtual tumour is composed of four types of cells which behave in four ways:

- *tumour cells* may proliferate at a rate proportional to their access to oxygen and nutrients modelled as a characteristic doubling time (${\upalpha}_{\mathrm{T}}$) and never until reaching a minimum time to divide ($\uptau >{\upbeta}_{\mathrm{T}}$) [20] (see equation (11)),
- tumour cells may undergo reversible hypoxia if their oxygen concentrations sink below a threshold (${\mathrm{h}}_{\mathrm{H}}$) for long enough ($\uptau >{\upbeta}_{\mathrm{H}}$) (see equation (14))
- *hypoxic cells* may irreversibly become *necrotic cells* if they are hypoxic for long enough ($\uptau >{\upbeta}_{\mathrm{N}}$) (see equation (15)),
- *stromal cells* may migrate in a random walk manner (controlled by ${\upmu}_{\mathrm{S}}$) or may be recruited close to the tumour (regulated by ${\mathrm{k}}_{\mathrm{S}}$), see equations (12)-(13).

Up until now the relationship between tumour and stromal tissue assumed in the model is cooperative-only. However, the stroma confers stiffness to the tumour matrix, thereby constraining growth. Since the model is built on a fixed lattice, there is a natural impossibility to translocate or divide if there is no empty space around and so, this constraint is built into the model, where h is the sum of empty spaces in the Moore neighbourhood of the cell in question﻿ (see equation (11)﻿ and (13)).

The model equations were written assuming that,

1. the tumour mass is composed of tumour tissue (in three states: viable, hypoxic, and necrotic) and stroma (containing fibroblasts, immune infiltrate, and vasculature);
2. all processes contain pure time delays, i.e. the cells have to spend a minimum time in the cell cycle before dividing; we avoid hereby the non-realistic instantaneous cell division;
3. hypoxia and necrosis appear sequentially following increasing exposure to oxygen deprivation;
4. hypoxia is reversible, providing oxygen levels are recovered;
5. stromal tissue has a purely synergistic biochemical relationship with tumour tissue, supplying oxygen and nutrients; and
6. the high stiffness of the stroma may limit tumour progression.

These simple assumptions are enough to demonstrate the morphological and biomechanical roles of the stroma in different tumour phenotypes.

#### Definition of oxygen distribution

Define now the simplest problem in which within a tumour space V, there is one single element of stroma carrying functional vasculature that serves as a source of oxygen located at the point *x*_so_{x_so_, y_so_} ⊂ V (see Fig. 2a). The equations of oxygen diffusion are based on the Fick’s law of molecular diffusion,

1 $$ \frac{{\mathrm{dP}}_{\mathrm{O}2}}{\mathrm{dt}}+\nabla \left(\mathrm{D}\cdot \nabla {\mathrm{P}}_{\mathrm{O}2}\right)={\mathrm{k}}_{\mathrm{R}}{\mathrm{P}}_{\mathrm{O}2}=\mathrm{R}, $$

where, ${\mathrm{P}}_{\mathrm{O}2}$ is the oxygen partial pressure and $\mathrm{D}$ and ${\mathrm{k}}_{\mathrm{R}}$ are the diffusion and oxygen uptake rate by the cells (Rate R) and $t$ is a time variable.

Assuming first that the diffusion process occurs much more rapidly than the accumulation of HIF1α and the structural changes at tissue level, we can approximate Eq. (1) to a quasi-steadystate $\left(\frac{\mathrm{d}{\mathrm{P}}_{\mathrm{O}2}}{\mathrm{d}\mathrm{t}}\approx 0\right)$. With this and assuming that the diffusion coefficient is independent of the location in the tissue $\left(\mathrm{D}\ne \mathrm{f}\left(\mathrm{space}\right)\right)$, we obtain,

2 $$ \mathrm{D}\cdot {\nabla}^2{\mathrm{P}}_{\mathrm{O}2}={\mathrm{k}}_{\mathrm{R}}{\mathrm{P}}_{\mathrm{O}2}. $$

Define P_O2_(*x*_*s*_) ∈ ℝ^2^ as the map of oxygen computed for the first single point (*x*_s_ ∈ V). Now let us define the boundary conditions as (1) Diritchlet boundary at the stromal cell, where the concentration of oxygen should match the oxygen concentration in blood giving the constant P_O2_(*x*_s_) = P_O2_^blood^ and (2) Neumann bounds at the stroma cell, where there is a maximum (∇P_O2_(*x*_s_) = 0), and oxygen decreases gradually until infinity ($\nabla {\mathrm{P}}_{\mathrm{O}2}\left(\infty \right)=0$).

There is no analytic closed-form solution of the Eq. (2) for the given boundary conditions when the number of stromal cells grow. However, a reasonable equation contained in the solution space should contain the terms,

3 $$ {\mathrm{P}}_{\mathrm{O}2,\mathrm{s}}={\mathrm{C}}_1\cdot {\mathrm{e}}^{-{\mathrm{C}}_2\left|| x-{x}_{\mathrm{s}}\right||}+{\mathrm{C}}_3\cdot {\mathrm{e}}^{+{\mathrm{C}}_4\left|| x-{x}_{\mathrm{s}}\right||}, $$

defined in the whole of V and where | |*x* − *x*_s_|| represents the Euclidean norm to the stromal cell in question. Now simply for the boundary conditions to be true, the expression ${\mathrm{C}}_3=0$ has to hold. This simplifies things significantly giving the expression

4 $$ {\mathrm{P}}_{\mathrm{O}2,\mathrm{s}}\left( x-{x}_{\mathrm{s}}\right)={\mathrm{C}}_1\cdot {\mathrm{e}}^{-{\mathrm{C}}_2\left|| x-{x}_{\mathrm{s}}\right||}, $$

for one single stromal cell. Applying the boundary conditions described before and assuming that the constant is a function of the uptake rate we obtain ${\mathrm{C}}_2=\sqrt{\frac{{\mathrm{k}}_{\mathrm{R}}}{9\mathrm{D}}}$ and ${c}_1={P}_{O2}^{blood}$ for a single stromal cell.

However, in our reality we consider a growing number of stromal cells, whereby the analytic solution of this is not generalisable. To extend this approach to the multi-source distribution, unlike for linear systems, the superposition principle cannot be applied. In this case, we will use a convenient approach in which the total equivalent distance will be calculated as the reciprocal sum of the distance to each stromal cell:

5 $$ {\left|\left| x-{x}_{\mathrm{s}}\right|\right|}_{\mathrm{eq}}=1/{\displaystyle {\sum}^{\mathrm{S}}\left(1/{\left|\left| x-{x}_{\mathrm{s}}\right|\right|}_{\mathrm{s}}\right)}. $$

Despite this being just a merely convenient approach, the approximation is valid to the degree of accuracy required as demonstrated in Additional file 1.

Lastly, we ought to find the function ${\mathrm{C}}_{2,\mathrm{eq}}=\mathrm{f}\left(\frac{{\mathrm{k}}_{\mathrm{R}}}{\mathrm{D}}\right)$ for the multiple source case, which we obtained empirically as

6 $$ {\mathrm{C}}_{2,\mathrm{eq}}=\sqrt{\frac{{\mathrm{f}}_{\mathrm{corr}}\cdot {\mathrm{k}}_{\mathrm{R}}}{9\cdot \mathrm{D}}} $$

with ${\mathrm{f}}_{\mathrm{corr}}\in \mathbb{R}$ and fixed value of ${\mathrm{f}}_{\mathrm{corr}}=400$ obtained by minimising the disparity between the analytic solution produced by Eq. (5) and the numeric solution computed with a Gauss-Seidel algorithm as further explained in Additional file 1. The Eqs. (4), (5), and (6) summarise in

7 $$ {\mathrm{P}}_{\mathrm{O}2,\mathrm{s}}\left( x-{x}_{\mathrm{s}}\right)={\mathrm{P}}_{\mathrm{O}2,\mathrm{s}}\cdot {\mathrm{e}}^{-\frac{20}{3}{\mathrm{k}}_{\mathrm{R}}^{{\textstyle \hbox{'}}}{\left|\left| x-{x}_{\mathrm{s}}\right|\right|}_{\mathrm{e}\mathrm{q}}} $$

which was taken as the final expression used for the simulations of this model, with k_R_'=√k_R_/D as the apparent oxygen uptake rate.

#### Definition of tumour growth

For the tumour cells, the oxygen uptake is directly proportional to the division rate. The proliferation constant is represented by a time constant ${\upalpha}_{\mathrm{T}}$ and a pure time delay ${\upbeta}_{\mathrm{T}}$, therefore the proliferation rate can be expressed as

8 $$ \frac{\Delta \mathrm{T}\left(\uptau \right)}{\Delta \uptau}\approx \frac{\mathrm{d}\mathrm{T}\left(\uptau \right)}{\mathrm{d}\uptau}=\frac{{\mathrm{P}}_{\mathrm{O}2}(x)}{\upalpha_{\mathrm{T}}}\cdot \mathrm{T}\left(\uptau -{\upbeta}_{\mathrm{T}}\right). $$

Now substituting the oxygen levels on (7) with the Eq. (8) and integrating between ${\displaystyle \int \frac{\mathrm{dT}\left(\uptau \right)}{\mathrm{T}\left(\uptau -{\upbeta}_{\mathrm{T}}\right)}={\displaystyle {\int}_{\uptau ={\upbeta}_{\mathrm{T}}}^{\uptau}\frac{{\mathrm{P}}_{\mathrm{O}2}(x)}{\upalpha_{\mathrm{T}}}\mathrm{d}\uptau}}$ we obtain

9 $$ \frac{\mathrm{T}\left(\uptau \right)}{\mathrm{T}\left({\upbeta}_{\mathrm{T}}\right)}={\mathrm{e}}^{\frac{{\mathrm{P}}_{\mathrm{O}2}(x)}{\upalpha_{\mathrm{T}}}\left(\uptau -{\upbeta}_{\mathrm{T}}\right)} $$

where ${\upbeta}_{\mathrm{T}}$ is the time at which the probability for transition is non-zero. This will be represented with a step function $\mathcal{H}\left(\uptau -{\upbeta}_{\mathrm{T}}\right)$ with $\mathcal{H}$ being a one dimensional Heaviside function. Now since the logical expression: $\mathrm{T}\left(\uptau \right)> T\left({\upbeta}_{\mathrm{T}}\right)\forall \uptau >{\upbeta}_{\mathrm{T}}$ holds, we invert the Eq. (9) to determine the fraction of cells that divided at any given time compared to the initial ones,

10 $$ {\mathrm{X}}_{\mathrm{T}\to 2\mathrm{T}}\left(\uptau \right)=1-\frac{\mathrm{T}\left({\upbeta}_{\mathrm{T}}\right)}{\mathrm{T}\left(\uptau \right)}=1-{\mathrm{e}}^{-\frac{{\mathrm{P}}_{\mathrm{O}2}(x)}{\upalpha_{\mathrm{T}}}\left(\uptau -{\upbeta}_{\mathrm{T}}\right)}\ \forall\ \uptau >{\upbeta}_{\mathrm{T}} $$

where ${\mathrm{X}}_{\mathrm{T}\to 2\mathrm{T}}\left(\uptau \right)$ symbolises the fraction of cells that divide.

Subsequently, if we take the continuous deterministic fraction of dividing cells just defined in Eq. (10) in discrete terms, it follows that for each single cell with oxygen level defined by its position, the probability for it to commit to the cell cycle is statistically proportional to the fraction of proliferating cells ($\Pr \left(\mathrm{T}\to 2\mathrm{T}\right)\propto {\mathrm{X}}_{\mathrm{T}\to 2\mathrm{T}}$). However, in this case this probability is truncated by the pure time delay (defined as a Heaviside step function) and the availability of space to divide, where the expression (10) substituting for Eq. (7) gives the final expression (11).

In turn, stromal cells may be recruited with probability (12) and move with probability (13). Lastly, tumour cells may become hypoxic and subsequently necrotic with probabilities governed by the age of cells and oxygen reach as described in (13) and (14).

The equations and a graphical display are summarised below and in Fig. 2, whereas the codes can be found in Additional file 2.

### Summary of equations

**Table:** 

Tumour	$\Pr \left(\mathrm{T}\to 2\mathrm{T}\right)=\left(1,-,{\mathrm{e}}^{-{\mathrm{e}}^{-\frac{20}{3}\cdot {\mathrm{k}}_{\mathrm{R}}^{{\textstyle \hbox{'}}}\cdot {\left|\left| x-{x}_{\mathrm{s}}\right|\right|}_{\mathrm{e}\mathrm{q}}}\cdot \left(\tau -{\beta}_{\mathrm{T}}\right)/\left({\upalpha}_{\mathrm{T}}\right)}\right)\cdot \mathcal{H}\left(\tau -{\beta}_{\mathrm{T}}\right),\kern0.5em \mathrm{f}\mathrm{o}\mathrm{r}\mathrm{h}\ne 0,$	(11)
Stroma	Recruitment $\mathrm{\triangle}\mathrm{S}\left(\mathrm{t}\right)={\mathrm{k}}_{\mathrm{S}}\cdot \mathrm{T}\left(\mathrm{t}\right)\cdot \mathrm{\triangle}\mathrm{t}$,	(12)
	Motility $\Pr \left({\mathrm{S}}_{0\to 1}\right)={\mu}_{\mathrm{S}}\cdot \Delta \mathrm{t}\cdot \mathrm{S}\left(\uptau \right)/\mathrm{T}\left(\uptau \right)$, for $\mathrm{h}\ne 0$,	(13)
Hypoxia	$\Pr \left(\mathrm{T}\to \mathrm{H}\right) = \mathcal{H}\left(\uptau -{\upbeta}_{\mathrm{H}}\right)\cdot \mathcal{H}\left({\mathrm{P}}_{\mathrm{O}2}-{\mathrm{h}}_{\mathrm{H}}\right)$	(14)
Necrosis	$\Pr \left(\mathrm{H}\to \mathrm{N}\right) = \mathcal{H}\left(\uptau -{\upbeta}_{\mathrm{N}}\right)$	(15)

The TSM was run stochastically at a time step of $\Delta \mathrm{t}=3\mathrm{h}$ on a square 2D lattice of 50×50 voxels. The initial conditions are described in Additional file 1. Once the probabilities have been calculated, the algorithm should check the events one by one, comparing a random number to their probability. The steps can be summarised as:

1. selection of time step $\Delta \mathrm{t}$,
2. update time,
3. randomly select each possible event and compare the probability of each event ${\mathrm{f}}_{\mathrm{e}}\left(\uptau, \mathrm{r}\right)$ to a random number,
4. update the state,
5. repeat 3–4 until all possible events have been checked,
6. repeat from 2 until simulation time is reached.

#### Parameter estimation model

The PEM transforms both 2Dimensional cell culture and 3Dimensional tissue slice culture biomarker expressions into the in-vivo parameter values of the TSM using a number of mathematical strategies graphically summarised in Fig. 2a. Briefly, the PEM is subdivided into five modules: growth curve module, immunohistochemistry (IHC) image processing module, heterogeneity module, Western blot module, and the oxygen-diffusion module. A mathematically formal description of these modules is provided below.

#### Growth curve module

**Fig. 3:**
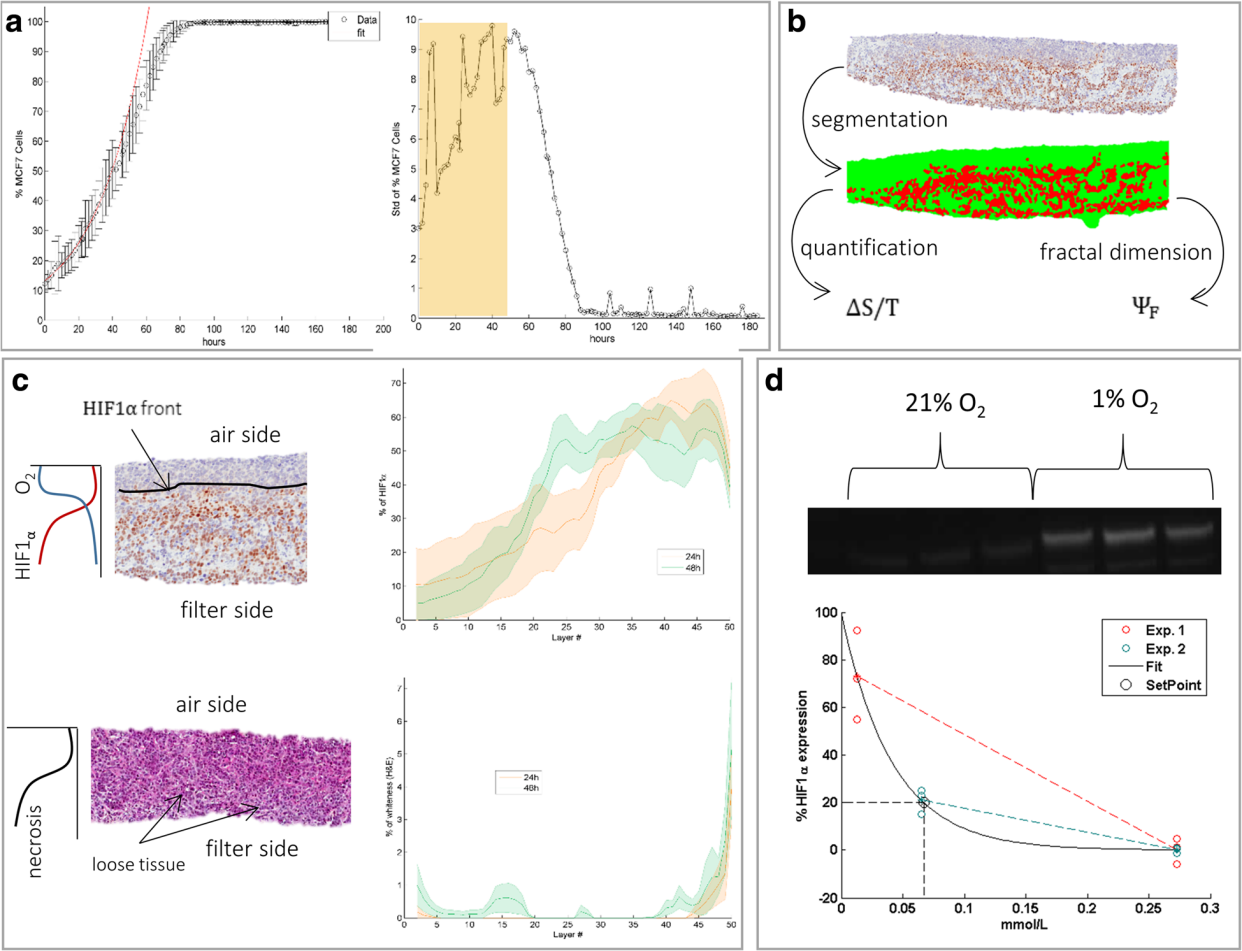
Results of parameter estimation from in-vitro data on the cell line MCF7. **a** 2D in-vitro cell growth from Incucyte data, as % of cell confluence (*left*) and standard deviation (*right*). They were used to calculate **α**_**T**_ and **β**_**T**_ respectively. **b** Illustration of **HIF1α** histopathology segmentation by colour deconvolution. The segmented patterns in *red* were quantified and the calculated the fractal dimension to estimate **μ**_**s**_ and **k**_**s.**_ **c** Example of **HIF1α** (*top*) and H&E (*bottom*) used to quantify the transversal distribution of hypoxia and necrosis (*right*). **d** Western blot data (n=3) showing the correlation of expression of **HIF1α** with atmospheric oxygen

Tumour parameters ${\upalpha}_{\mathrm{T}}$ and ${\upbeta}_{\mathrm{T}}$ will be calculated from in-vitro cell confluence assays on the cell line MCF7. We use the mean of the populations of cells as the expected value for a quasi-exponential cell growth with constant $1/{\upalpha}_{\mathrm{T}}$ (see Fig. 3a, fit) and the standard deviation as a measure of the diversity in the delay for the cells to commit to the cell cycle (${\upbeta}_{\mathrm{T}}$). Let ${\mathrm{C}}_{\mathrm{n}}\left(\mathrm{t}\right)$ be the % of confluence, $\mathrm{t}$ the time measured, and ${\upsigma}_{\mathrm{Cn}}\left(\mathrm{t}\right)$ the standard deviation. We can then define our parameters as,

16 $$ {\upalpha}_{\mathrm{T}}={\left(\frac{1}{\mathrm{t}}\right)}^{-1}\times \mathrm{L}\mathrm{n}\left({\mathrm{C}}_{\mathrm{n}}\right),\mathrm{and} $$

17 $$ {\upbeta}_{\mathrm{T}}={\upalpha}_{\mathrm{T}}\cdot \mathrm{L}\mathrm{n}\left({\displaystyle {\int}_{\mathrm{t}=0\mathrm{h}}^{\mathrm{t}=50\mathrm{h}}{\upsigma}_{\mathrm{Cn}}\left(\mathrm{t}\right)\cdot \mathrm{dt}}\right), $$

where the linear matrix product in Eq. (16) was solved by Gaussian elimination. All units are in hours.

The results for both parameters for the cell line MCF7 are ${\upalpha}_{\mathrm{T}}=9.23\mathrm{h}$ and ${\upbeta}_{\mathrm{T}}=18.17\mathrm{h}$, which together sum up to 27.4 h. Surprisingly, this value is very close to the doubling times reported in the literature (20–40 h [21]) and somewhat higher to the doubling time calculated from the same data set (20.64 h).

#### Immunohistochemistry (IHC) module

We now concentrate on the spatial distribution of hypoxia biomarkers to estimate oxygen diffusion parameters. IHC was carried out on paraffin embedded tissue slice sections as has been previously described in Davies et al. [22]. To calculate delays in the onsets of hypoxia and necrosis we used tissue culture experiments on thirteen different slices, which after isolation, sectioning, and staining we quantified digitally and discretised evenly with the so-called Tissue Culture Profiling (TCP) algorithm (as described in the Additional file 1), giving the results shown in Fig. 3c. Judging by the mismatch between the two curves at 24 h and 48 h (see Fig. 3c) there is a delay in the onset of HIF1α, also previously described as inherent to the cell biology [23]. We calculated these delays in the onset of hypoxia with the so-called threshold-based method which utilises a binary onset of hypoxia at a certain absolute oxygen level threshold value, although other methods have been explored as summarised in [11].

This method utilises the absolute curve shift between the curves at 24 and 48 h for 20% expression of HIF1α and 10% of necrosis as the standards to calculate the delays. The parameters can be calculated with

18 $$ {\upbeta}_{\mathrm{H}}=\frac{50}{\left|\mathrm{nl}\left(20\%\mathrm{Hyp},\ 24\mathrm{h}\right)-\mathrm{nl}\left(20\%\mathrm{Hyp},\ 48\mathrm{h}\right)\right|}24\mathrm{h},\ \mathrm{and} $$

19 $$ {\upbeta}_{\mathrm{N}}=\frac{50}{\left|\mathrm{nl}\left(10\%\mathrm{Nec},\ 24\mathrm{h}\right)-\mathrm{nl}\left(10\%\mathrm{Nec},\ 48\mathrm{h}\right)\right|}24\mathrm{h}, $$

where ${\upbeta}_{\mathrm{H}}$ and ${\upbeta}_{\mathrm{N}}$ are the delays of hypoxia and necrosis for the threshold-based method. Their values are ${\upbeta}_{\mathrm{H}}=139\mathrm{h}$ and ${\upbeta}_{\mathrm{N}}=184\mathrm{h}$, which correspond to 43 μm and 32 μm of curve shift respectively.

#### Heterogeneity module

The heterogeneity module uses imaging data to output a measure of stromal heterogeneity, which we define based on the fractal dimension of the 2D image. We estimate fractal dimensions via a counting-box algorithm, which counts boxes of decreasing sizes needed to cover the region of interest of our image. The total count of boxes ($\epsilon$) is then fitted to a line in a double-logarithmic scale and extrapolating it to size zero. Thus,

20 $$ {D}_0= l i{m}_{\in \to 0}-\frac{ log N\left(\epsilon \right)}{log\left({\in}^{-1}\right)}. $$

From the fractal dimensions, a single value of the heterogeneity has been taken into account (assuming for a 2D image), where the heterogeneity is a deviation from the “integer” dimension, thus

21 $$ {\varPsi}_F=2-{D}_0, $$

where ${\Psi}_F$ represents the heterogeneity calculated from the fractal dimension.

Finally, the stromal parameters are described directly by the total quantification and by the heterogeneity factor, as

22 $$ {k}_S=\frac{d\left( S/ T\right)}{10\cdot dt},\  and $$

23 $$ {\mu}_S=\frac{d\left({\varPsi}_F/\left( S/ T\right)\right)}{ d t}, $$

where ${\Psi}_{\mathrm{F}}$ is again the fractal heterogeneity and S/T is the stroma to tumour ratio, usually expressed as a percentage. Note that T refers only to the tumour fraction and not the total, hence $\mathrm{T}=\left(\mathrm{Total}-\mathrm{S}\right)$. The value of 10 is a convenient number added to counterbalance the error introduced by the analysis of a cross-section in lieu of the whole tissue culture in-vivo.

Now accounting for the available data we obtain,

24 $$ {\mathrm{k}}_{\mathrm{S}}\approx \frac{\Delta \mathrm{S}/\mathrm{T}}{\Delta \mathrm{t}}\approx \frac{\mathrm{S}/\mathrm{T}\left(48\mathrm{h}\right)-\mathrm{S}/\mathrm{T}\left(24\mathrm{h}\right)}{10\cdot \left(48-24\right)},\ \mathrm{and} $$

25 $$ {\upmu}_{\mathrm{S}}\approx \frac{\Psi_{\mathrm{F}}\left(24\mathrm{h}\right)/\mathrm{S}/\mathrm{T}\left(24\mathrm{h}\right)-{\Psi}_{\mathrm{F}}\left(0\mathrm{h}\right)/\mathrm{S}/\mathrm{T}\left(0\mathrm{h}\right)}{24-0} $$

for both parameters respectively which results in values of 0.064 h^−1^ (Str/T)^−1^ and 0.0031 h^−1^ (Str/T)^−1^. This calculation has been summarised in Fig. 3b.

#### Western blot module

To find the oxygen concentration at which HIF1α is expressed, the MCF7 cell line was cultivated in-vitro at different oxygen concentrations (1, 5, and 21%) and then Western blots [24] were run for HIF1α. Standard Western blot protocols were used, HIF1α (#610959, BD Biosciences, 1:250 dilution). The intensity of the chemoluminescense is proportional to the amount of HIF1α in the sample (see Fig. 3d). The experiments were done on two separate occasions: first 1 and 21% O_2_ atmospheres were used, then 5 and 21% O_2_ with a total of 12 measurable points. Since the chemiluminescent exposure may have been slightly different, the sample results have been linearly normalised for the reference average of 21% O_2_.

The results show a fair exponential distribution (as expected) with formula

26 $$ \% H I F1\alpha =100\cdot {e}^{-24\cdot {C}_{O2}},{C}_{O2}\kern0.5em \mathrm{in}\ \mathrm{mmol}/\mathrm{L},\ \mathrm{and} $$

for which an arbitrary expression of 20% of HIF1α would need around 0.064 mmol/L of oxygen in tissue or around 5% of atmospheric oxygen in culture (see Fig. 3d). The estimation has been done with a naïve-pooled algorithm with the 12 points depicted in Fig. 3d.

#### Oxygen diffusion module

In this module, we attempt to calculate oxygen diffusion parameters as described in Eq. (2) by means of a tissue slice experiment. The geometry of the slice leads to the one dimensional resolution of Eq. (2) solved in Cartesian coordinates, with Dirichlet boundary conditions for both sides of ${\mathrm{P}}_{\mathrm{O}2}\left({\mathrm{x}}_{\mathrm{filter}}\right)=0.28\;\mathrm{mmol}/\mathrm{L}$ and ${\mathrm{P}}_{\mathrm{O}2}\left({\mathrm{x}}_{\mathrm{air}}\right)=$ $1.19\;\mathrm{mmol}/\mathrm{L}$, being these calculated from themodynamic equilibrium expressions (see section Additional file 1). These quantities are comparable to the cava return veins and alveolar concentrations [25]. The approximate analytic expression results in

27 $$ {\mathrm{P}}_{{\mathrm{O}}_2}\left(\mathrm{x}\right)={\mathrm{P}}_{{\mathrm{O}}_2}\left({\mathrm{x}}_{\mathrm{air}}\right)\cdot \frac{ \cosh \left({\mathrm{k}}_{\mathrm{R}}^{\hbox{'}}\cdot \mathrm{x}\right)}{ \cosh \left({\mathrm{k}}_{\mathrm{R}}^{\hbox{'}}\cdot {\mathrm{x}}_{\mathrm{air}}\right)} $$

whereby ${\mathrm{k}}_{\mathrm{R}}^{\prime }=\sqrt{{\mathrm{k}}_{\mathrm{R}}/\mathrm{D}}$ and $\mathrm{x},{\mathrm{x}}_{\mathrm{air}}$ are the 1D positions in the transversal direction of the slice. Note that in this case the origin of coordinates was set at the filter (${\mathrm{x}}_{\mathrm{filter}}=0$). A two dimensional version of the algorithm was also discussed in [11].

#### Summary of results of the PEM

The complete parameter set has been estimated for the cell line MCF7 only, which results are presented in Table 1. To expand the use of the model and test the formation of complex stromal patterns, we decided to explore the cell lines Calu3 and Calu6, which demonstrate very different stromal morphological features between them. However, data availability is limited for the last two cell lines. To solve this problem, the values of parameters ${\beta}_H,{\beta}_N,{k}_R^{\prime },\mathrm{and}\;{h}_H$ were assumed to be the same as for MCF7, whereas the values of ${\upalpha}_{\mathrm{T}},{\upbeta}_{\mathrm{T}},{\mathrm{k}}_{\mathrm{S}},\mathrm{and}\;{\upmu}_{\mathrm{S}}$ were estimated using the ratio ${\upalpha}_{\mathrm{T}}/{\upbeta}_{\mathrm{T}}$ unchanged but corrected for the doubling times of the cell lines (35 h for Calu3 and 26 h for Calu6 [26]) and the whole tumour histological images to correct for the stromal recruitment. For the codes and examples on the PEM see Additional file 3.

**Table 1:** References for the algorithms contained in each module used to calculate each parameter of the model for the cell lines MCF7, Calu3, and Calu6

Parameter	Units	Value		
		MCF7	Calu3	Calu6
***α***_***T***_	h	9.23	11.4	8.7
***β***_***T***_	h	18.17	23.2	17.24
***β***_***H***_	h	139	139	139
***β***_***N***_	h	184	184	184
***k***_***R***_'	cm^−1^	185	185	185
***h***_***H***_	mmol/L	0.064	0.064	0.064
***k***_***S***_	h^−1^ (Str/T)^−1^	0.0031	0.017	0.0041
***μ***_***S***_	h^−1^ (Str/T)^−1^	0.0013	0.0013	0.0013

## Results & discussion

Once the algorithms have been presented and the parameters estimated, it follows to analyse the potential of their tumour-stromal model in the different phenotypes.

### Simulations on complete parameter set from parameter estimation model

**Fig. 4:**
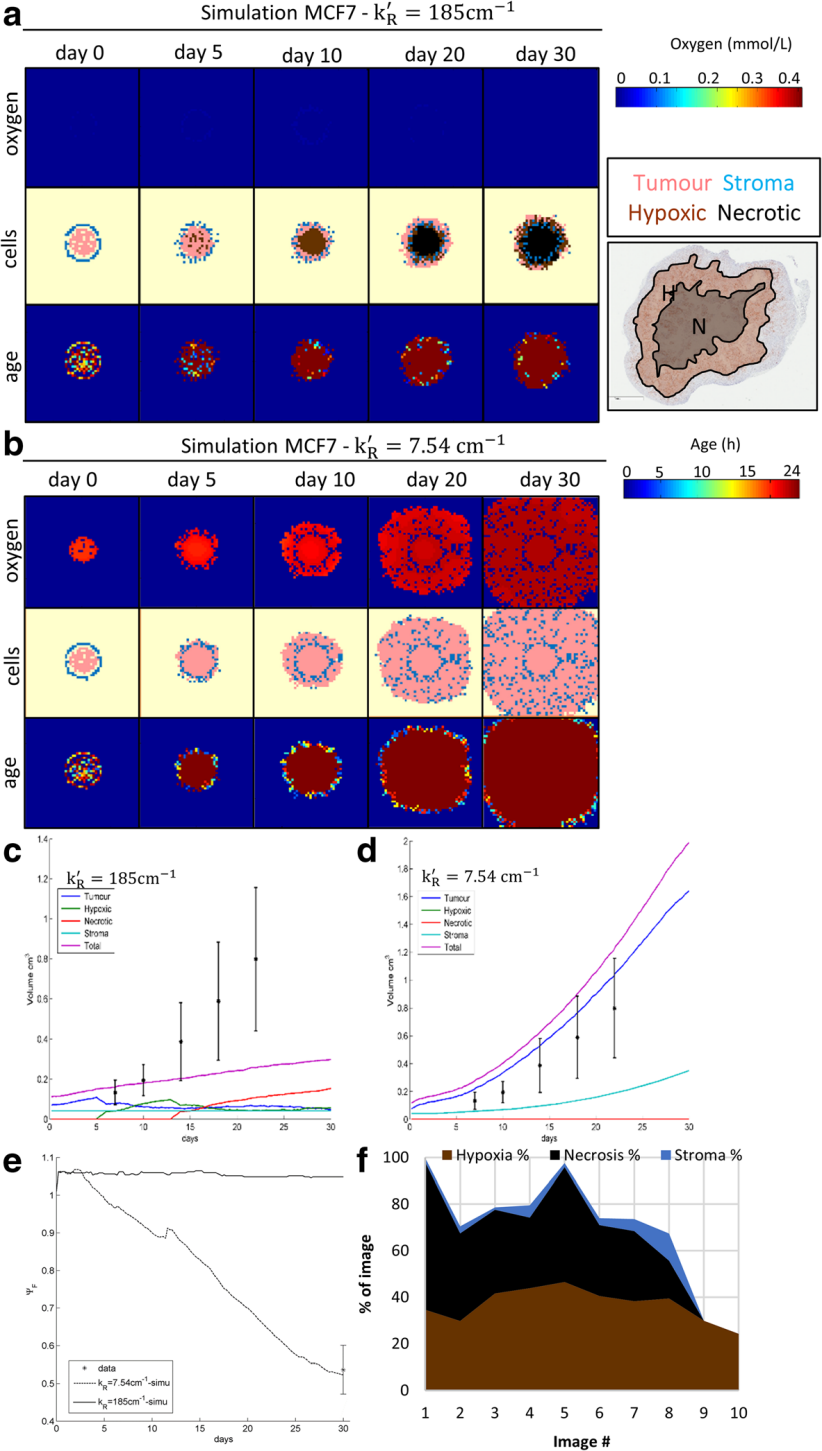
Thirty day simulation for the parameters estimated on Table 1 for the cell line MCF7 in 2D. **a** Results with ***k***_***R***_^'^ = **185** ***cm*** ^− 1^. *Top row* shows oxygen heat maps (scale 0–0.46 mmol/L) on different days, middle row shows the cell distribution and bottom row heat maps of age (scale 0–24 h). Picture on the *right* is a sample IHC for MCF7 stained for HIF1α with necrotic and hypoxic regions outlined. **b** results with ${\boldsymbol{k}}_{\boldsymbol{R}}^{\hbox{'}}=\mathbf{\mathsf{7}}.\mathbf{\mathsf{54}}\boldsymbol{c}{\boldsymbol{m}}^{-1}$. **c**-**d** Volumetric results for ***k***_***R***_^'^ = **185** ***cm*** ^− 1^ and ${\boldsymbol{k}}_{\boldsymbol{R}}^{\hbox{'}}=\mathbf{\mathsf{7}}.\mathbf{\mathsf{54}}\boldsymbol{c}{\boldsymbol{m}}^{-1}$ respectively. **e** Calculated values of heterogneity. **f** Observed surface area coverage of necrosis, hypoxia and stroma as determined by expert digital pathology

Using the parameters obtained from the in-vitro platforms for MCF7 summarised in Table 1, we have made simulations to build Fig. 4a. In this figure, we observe that the necrotic and hypoxic regions appear concentrically as it does in reality (see IHC image on Fig. 4a and Ref. [27]); the oxygen levels are very low; and the viable rim is of almost constant thickness, which is consistent with numerous observations [28]. All these properties are congruent with the literature and our data; and are futher equivalent to those of an avascular tumour.

However, the values reported for MCF7 obtained from the fitting of other in-vivo models [19] resulted to be ${k}_R^{\prime }=7.54 c{m}^{-1}$ which is 24.5 times smaller than that reported in the here estimated in-vitro system. This –as reported in by the authors in Ref. [19] - is due to the increased oxygenation proportionated by the angiogenenic vessels. If we now simulate the same system changing the value of ${k}_R^{\prime }$, we observe that there is no apparent difference in the distribution of stroma. However, the viability of the tissue has changed, as the whole tissue is well perfused and viable (Fig. 4a). The actual linear oxygen reach now would have increased to 6.5 mm. This does not reflect the real tissue distribution as shown by the IHC (Fig. 4f) but will be useful to emulate a number of clinically possible scenarios as we will see later on.

The model predictions for ${k}_R^{\prime }$ value of $185 c{m}^{-1}$ fail to represent the growth rate accurately, although the estimations are within a close range to the data (see Fig. 4d), whereas for the value of $7.54 c{m}^{-1}$ the predicted growth mimics almost perfectly the growth rate (see Fig. 4d). The overall growth rates differ by 82.4% at the end of the study, which is a remarkable difference (Fig. 4c-d). The moderate stromal growth (~25% at 0 days to 17.5% and 11.7% at 30 days) is slightly higher than that of the real observations, where the proportion of the stroma at the end of the study lies around $4.1\%\pm 3.4\%$ at day 30 (n=8, Fig. 4f). It is important to point out that those values were calculated by manually outlining the visual stromal regions on images stained for HIF1α. This means that the values may (and probably will) vary greatly when stained for activated stroma (αSMA). However, due to the lack of time and staff those experiments were not conducted. The predicted proportions of hypoxia (24.3%) and necrosis (48.6%) are likewise within the ranges of the observed values with means of $36.8\%\pm 7.1\%$ (*n*=10) and $36.7\%\pm 14.1\%$ (*n*=8) respectively (Fig. 4f).

It is interesting that the modification of the oxygen uptake rate alone is sufficient to simulate vastly different possible clinical scenarios, however none of those is completely congruent with both the growth rate and stromal distribution phenotypes (see Fig. 4). On the one hand, a low ${k}_R^{\prime }$ will provide with high rates of proliferation to the cells with space to proliferate, which is consistent with the overall growth rates observed. On the other hand, we see a large necrotic core forming in the tumour, which is indicative that ${k}_R^{\prime }$ is actually a function of the tumour position with radial geometry.

### Modelling the different phenotypes: Calu3 and Calu6

**Fig. 5:**
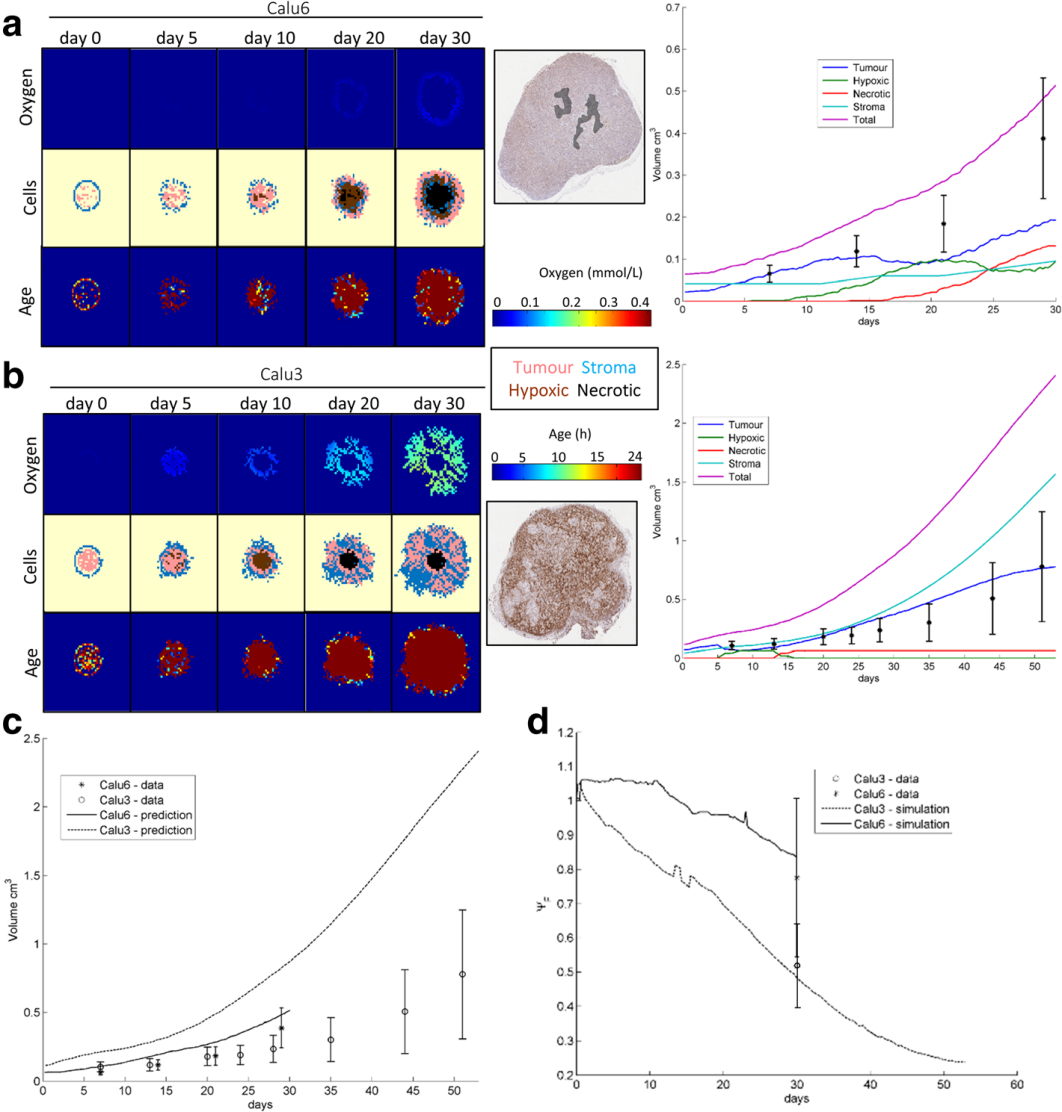
Tumour-stroma model and results. **a** Tissue pattern results for oxygen, age and cells with example IHC tumour cross-section for Calu3. **b** Tissue pattern results for oxygen, age and cells with example IHC tumour cross-section for Calu6. **c** Growth curve results. **d** Evolution of heterogeneity

The parameters estimated for Calu3 and Calu6 (Table 1) were used in the equations (11)-(15) to simulate the results shown in Fig. 5. Firstly, Calu6 (Fig. 5b) shows a very similar profile as the one shown on the MCF7 cell line (owing to the similar parameter values, compare Table 1), which again is congruent with reality, as Calu6 and MCF7 present with similar histological profiles.

However, when we look at Calu3 the situation changes. Note that the only parameters that differ are the proliferation rates ${\upalpha}_{\mathrm{T}}$ and ${\upbeta}_{\mathrm{T}}$, and the stromal recruitment ${\mathrm{k}}_{\mathrm{S}}$ (Table 1). Now, if we look at the oxygen maps, these have changed significantly. The growing representation of the stromal tissue raises oxygen levels keeping the tissue viable. We observe this in the increased viable rim on Fig. 5a-b. The heterogeneity of Calu3 seems to be also lower, breaking with the concentric symmetry observed in the other two cell lines Calu6 and MCF7.

After validation of the patterns observed for Calu3 and Calu6 it is worth examining the volumetric growth rates predicted by the model (see Fig. 5c). The key observations here are that the proportion of stroma in Calu3 is significantly higher; at 15 days the hypoxic fraction has disappeared in Calu3 while it grows steadily for Calu6; and consequently the necrosis fraction reaches a plateau for Calu3 whereas for Calu6 it continues expanding (Fig. 5a-b).

Tumour volumes calculated with the TSM are higher than the data (Fig. 5c), although within similar ranges. Most importantly, the global growth speed of either in-vivo model is incongruent with the data, because the simulated Calu3 grows faster than simulated Calu6, which is in conflict with empirical observations.

In terms of stroma, when Calu3 and Calu6 tumour xenografts are analysed by IHC we observe that not only the amount of stroma is significantly higher in Calu3, but also the Mean Vascular Density (MVD), which negatively correlates to the amount of necrosis in these images, see Additional file 1). The quality of the vessels observed in average was similar in both models with no significant differences in vessel thickness, vascular area, lumen area or vessel perimeter (see Additional file 1).

The simulations produced by the original parameter set (${\mathrm{k}}_{\mathrm{R}}^{\prime }=185\mathrm{c}{\mathrm{m}}^{-1}$) fail to reproduce the heterogeneity at the end of the study for MCF7, whereas for the adapted parameter set (${\mathrm{k}}_{\mathrm{R}}^{\prime }=7.54\mathrm{c}{\mathrm{m}}^{-1}$) the heterogeneity values are very close to the data (Fig. 4e). Moreover, the values of heterogeneity observed in the simulations for Calu3 and Calu6 mirrors almost perfectly the data (Fig. 5d). All trends are downwards with the progression of time, which is consistent with the visual inspection of the images and the general rise in entropy of the distribution of stromal cells (Fig. 5a-b and Fig. 4a-b). Interestingly, the evolution of heterogeneity for the adapted parameter set for MCF7 corresponds to the one observed for Calu3.

Simulations with parameter sets for both cell lines show remarkable similarities between the tumour, stroma, necrotic, and hypoxic cell distributions and the tumour histopathology observed for Calu6 (Fig. 5a) and Calu3 (Fig. 5b) when grown in-vivo. It is notable that the viable rim in each simulated phenotype varies significantly, as demonstrated by the oxygen distribution maps (Fig. 5a-b, top row). However, results on the total tumour volume show that the growth dynamics of the cell line Calu6 are well captured (Fig. 5c), whereas the cell line Calu3 appears to grow faster in the model simulations than in reality (Fig. 5c). This effect reveals that the tumour-stroma relationship might not only be synergistic by providing growth factors and nutrients to the tumour, but it might actually constraint its growth mechanically (fibrous stroma confers rigid extracellular matrices with lesser room for cell proliferation [29, 30]), and chemically (cytotoxic components of the immune infiltrate play an active role in the retardation of tumour growth [31]).

Lastly, a comprehensive analysis of various simulation settings in terms of time step, lattice size, or lattice dimensionality (3D) will be an interesting exercise which will provide with other insights of the TSM. Especially interesting will be the usage of a cell resolution lattice and a sufficiently short time step to properly capture diffusion of macromolecules and changes in cell polarity. These computations require high specification hardware and computation time.

## Conclusions

The PEM was a useful platform to generate in-vivo-relevant parameter values from in-vitro data; nevertheless its usage relies upon the availability of the in-vitro assays mentioned above. To circumvent this, we proposed a feasible parameter extrapolation method which resulted valid and may be useful to other scientists. The results generated by the PEM-calibrated TSM for the two cell lines Calu3 and Calu6 demonstrate that the tumour-stroma mutually-positive relationship assumed here does not represent both the stromal patterns and growth rates simultaneously. This means that while the stroma constrains growth by limiting space to grow, these effects do not explain the slower growth rate of Calu3 (Fig. 5c). Experimentally we observe an increase in thickness of the viable tumour rim of the Calu3 model, which is explained by the exacerbated blood vessel-carrying stromal recruitment described with the TSM. Overall, these models suggest that at a tissue level, the stroma limits tumour growth, and at a molecular level, the stroma fosters tumour viability. Empirically, it has been shown in-vitro that stromal components provide growth-limiting molecular signals in the form of cell-sequestering factors (not built into our model) [1], which may explain the disparity in predicted and observed tumour growth.

Our in-vitro-calibrated mathematical models describe aspects of in-vivo tumour biology extrinsic to the tumour cell, which may provide a valuable addition to existing predictive models, such as cancer pharmacokinetic-pharmacodynamic-efficacy models developed using cell line-derived xenograft models. This adds another dimension to simplistic tumour models which may allow us to better predict the effect of drugs in morphologically-complex clinically-relevant pathophysiologies [3, 9].

## Additional files

Additional file 1: Supplementary material: This file contains additional information on the experiments conducted and the raw results as well as algorithmic detail of the scripts that quantify the distribution of IHC stain within a tissue culture, and the calculation of the oxygen concentrations at either interface of the tissue slice. (PDF 927 kb)

Additional file 2: mainTSM_Code: Main script for the Tumour-Stroma Model (TSM). This script is a stand-alone script that simulates the model given certain parameter values and initial conditions. (CPP 9.34 kb)

Additional file 3: PEM_Code: Compressed folder containing the codes for the Parameter Estimation Model algorithms and example figures. These scrips are stand-alone and contain all the necessary information to replicate the data in the manuscript. (7Z 8793 kb)

## Abbreviations

H&E: Hematoxylin and eosin
HIF1α: Hypoxia inducible factor 1α
IHC: Immunohistochemistry
MVD: Mean vascular density
PEM: Parameter- estimation model
SM: Supplementary materials
TCP: Tissue culture profiling algorithm
TSM: Tumour-stroma model
