# Erratum to: Multiscalar cellular automaton simulates in-vivo tumour-stroma patterns calibrated from in-vitro assay data

**DOI:** 10.1186/s12911-017-0492-7

**Published:** 2017-06-23

**Authors:** J. A. Delgado-SanMartin, J. I. Hare, E. J. Davies, J. W. T. Yates

**Affiliations:** 1 Modelling and Simulation, Oncology IMED DMPK, AstraZeneca, Li Ka Shing Centre, Cambridge, CB2 0RE UK; 2 Bioscience, Oncology IMED, AstraZeneca, Li Ka Shing Centre, Cambridge, CB2 0RE UK; 3 Physics Department, University of Aberdeen, Aberdeen, Scotland; 4 GSK R&D Centre, Gunnels Wood road, Stevenage, SG1 2NY UK

## Erratum

Upon Publication of the original manuscript [1] discrepancies were highlighted in eqs. (13) and (14). These errors occurred due to typesetting errors and have since been corrected in the original PDF.
