# Supplementary material for: Multiscalar cellular automaton simulates in-vivo tumour-stroma patterns calibrated from in-vitro assay data
Source: BMC Med Inform Decis Mak. 2017 May 30;17:70. doi: 10.1186/s12911-017-0461-1 (PMC5450227; doi:10.1186/s12911-017-0461-1)
Supplement: Supplementary file 1 — Supplementary material: This file contains additional information on the experiments conducted and the raw results as well as algorithmic detail of the scripts that quantify the distribution of IHC stain within a tissue culture, and the calculation of the oxygen concentrations at either interface of the tissue slice. (PDF 927 kb) [file 12911_2017_461_MOESM1_ESM.pdf]

Supplementary material of the manuscript:

Multiscalar cellular automaton simulates in-vivo tumour-stroma patterns calibrated with in-vitro assay data

by

Delgado-SanMartin J, Hare JI, Davies EJ, and Yates JWT

Submitted to BMC Medical Informatics and Decision Making in 2016

S1 CALCULATION OF INITIAL CONDITION

To define the initial condition, we first need to perform some simple arithmetic. If we design the whole grid to be of total volume V_{WG} and grid size n_{WG} the aspect ratio can be calculated as

$$AR = \left(\frac{V_{WG}}{n_{WG}^2}\right)^{1/2}, \quad (S1)$$

which assigning values to $V_{WG} = 2\ cm^3$ in volume and grid size of $n_{WG} = 50\ voxels$ the aspect ratio would result in

$$AR = 0.02828\ cm/voxel, \quad (S2)$$

which makes every voxel measure $283\ \mu m$. Assuming that the average diameter of a cancerous epithelial cell is around $30\ \mu m$, its volume equals $4.5 \cdot 10^{-9}\ cm^3/\ cell$, and thence each voxel contains $1.8 \cdot 10^5$ cells. This was used in Table S1 to change the units of the parameter k'_R.

With the former and knowing that the size of the initial implant is 0.1ml the initial conditions are summarised in Table S 1 for the three cell lines in question.

Table S 1: metrics of the initial injection for all cell lines tested

Cell line	# cells	Volume cells	Confluence
	-	cm^3	%Volume
MCF7	$5 \cdot 10^6$	0.07	70
Calu3	$5 \cdot 10^6$	0.07	70
Calu6	10^6	0.014	14

S2 Tissue culture profiling (TCP) algorithm

As commented in the main paper, tissue cultures have been characterised spatially to calculate a number of parameters. For the spatial discretisation of the slices we chose a method based on the physical properties in terms of the oxygen distribution from the environment into the tissue.

In brief the algorithm will be described hereafter. Following registration of the image and export into a tif file from ImageScope, the image is processed in Matlab 13b. In short, the method consists of morphological transformations to simplify the image and orient it in the direction of the oxygen profile, followed by the identification of the biomarkers of interest and their quantification in spatially discretised bands. The pseudo-code description of the algorithm is described in Table S 2.

Table S 2:pseudo-code image processing algorithm for the tissue slices

Step 1	Image registration: .tif file loaded onto Matlab (I)	$I \equiv (n, m, 3) \in [0,255]^3 \in RGB$
Step 2	Identification of slice contour: first the image (I) is transformed (T) into grey scale (G) which is then segmented (IS) with a variable threshold criterion (th_{BW}), giving a black and white image (Bw). This image is then morphologically modified by opening (with threshold th_{open}) and closing it (with threshold th_{close})	$T(I) \rightarrow G \in [0,1]^2$; $IS(G, th_{BW}) \rightarrow Bw \in \{0,1\}^2$; $T(Bw, \rho_{open}, \rho_{close}) \rightarrow Bw_m$
Step 3	Rotation (I_{Rot}): the algorithm calculates from the centroid ($c = (c_1, c_2)$) the closest boundary point (b_1), and displays the orthogonal lines connecting these two points. These lines suggest to the user an angle of rotation to axially orientate the image, however, depending on the roughness of the boundary, this suggestion needs verification. The algorithm offers to manually select the boundary point (b_2) giving the correct angle of rotation (θ_{Rot}).	$\theta_{Rot} = acos\left(\frac{\overrightarrow{c(c_1,0)} \cdot \overrightarrow{cb_2}}{\lVert\overrightarrow{c(c_1,0)}\rVert \cdot \lVert\overrightarrow{cb_2}\rVert}\right)$ $I_{Rot} = \begin{bmatrix} cos\theta_{Rot} & -sin\theta_{Rot} \\ sin\theta_{Rot} & cos\theta_{Rot} \end{bmatrix} \times I$
Step 4	Selection of air side. A yes/no question selects	$\theta_{flip} \in \{0,1\}$

	select the flipping angle (θ_{flip})	
Step 5	Colour deconvolution (CD), as described[1].	$CD(I_{Rot}) \rightarrow I_1, I_2, I_3 \in \mathbb{R}^2$
Step 6	Image segmentation (IS) is done by binarising at 3 different levels with thresholds ($th_{weak}, th_{medium}, th_{strong}$)	$IS(I_1, \rho_i) \rightarrow I_{1,seg} \in \{0,1\}^2$, for i = {weak, medium, strong}
Step 7	Spatial discretisation (SD) is performed by dividing the transversal dimension into $n_{layers} \approx 50$ layers. Top and bottom boundaries have been interpolated with a number of matching transversal points ($n_{tp} \approx 100 - 200$)	$SD(Bw, n_{layers}, n_{tp}) \rightarrow band_1, band_2, \dots, band_{nlayers} \in \{0,1\}^2$
Step 8	Quantification, the segmented image is quantified in each of the bands	$q_i = \sum \left(I_{1,Seg}(band_i)\right)$, for $i = [1, n_{layers}] \in \mathbb{N}$

Differently processed images illustrate the different levels of sophistication in the segmentation (Figure S 1 A-C). The four level segmentation (Figure S 1 B) shows a significant improvement in granularity of the data visualisation compared to the binary discretisation (Figure S 1 B). Finally, after being quantified the results may be displayed via a line or bar plot (see Figure S 1 D).

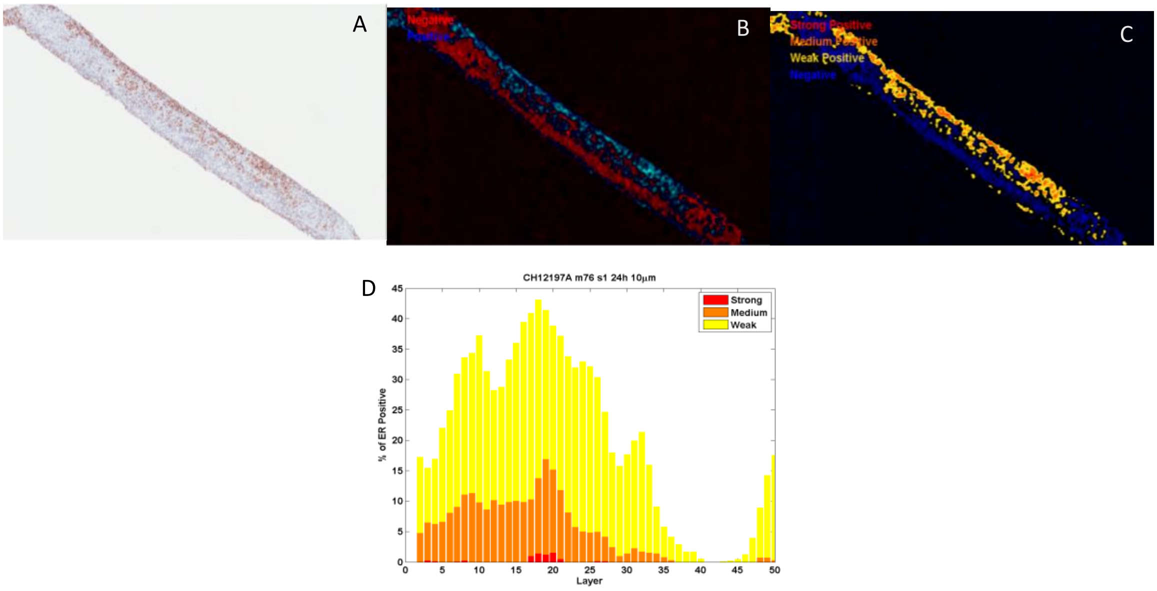

Figure S 1: example of image registration and results of the image processing algorithm for tissue slices stained for ERα (the same technique was used for HIF1α). A) raw image, B) image binarily segmented, C) image segmented in three levels D) example bar plot of one single slice divided into three levels.

S3 SUPPLEMENTARY INFORMATION ON THE OXYGEN-DIFFUSION MODULE

In the parameter-estimation model the parameters referring to the oxygen distribution and consumption are calculated by means of tissue cultures with asymmetric oxygen supply (one surface is exposed to medium and one to air). However, to calculate the concentrations of oxygen on either side we applied Henry's law [2],

$$P_{O2} = k_{H,O2} \cdot x_{O2}, \tag{S3}$$

where $k_{H,O2}$ is the Henry's constant dependent on the nature of the solute, solvent, temperature, and pressure. For our system, Henry's constant is $k_{H,O2} = 134\frac{mmHg}{mmol/L}$ (modified units) [3]. Now, calculating saturations of oxygen in the medium, the oxygen saturation at 37°C yields 7mg/L, equivalent to 37.6 mmHg. Now, there are two oxygen transfer systems: air-medium and medium-slice. The relative rate of mass transfer will provide the appropriate boundary conditions. Nevertheless, the time scale analysis of these processes reveal that the medium-slice system is the rate liming step (hours versus minutes). Another argument that advocates for this time scale analysis is that, in the cultures the exposed surface of medium to the air is much larger compared to the surface of the tumour slice (7.5-8.5 cm^2 and 1-2cm^2 respectively). Through this medium-air interface is where the oxygen exchange occurs and hence equilibrium is quickly reached. Consequently, with aqueous oxygen concentration being almost constant throughout the experiment, the Dirichlet boundary conditions for both sides can be written as,

$$\mathrm{P_{O2,filter}} = 37.6\ \mathrm{mmHg} \text{ or } 0.28\ \mathrm{mmol/L}, \tag{S4}$$

$$\mathrm{P_{O2,air}} = 159.6\ \mathrm{mmHg} \text{ or } 1.19\frac{\mathrm{mmol}}{\mathrm{L}}, \tag{S5}$$

As it was described in the main paper.

Now to calculate the values of oxygen distribution parameter $\mathrm{k'_R}$, we used a 1D and 2D spatial distribution models as described in the main paper and in [4]. The results shown in Table S 3 illustrate that there are no major differences between the slices across time, with averages of 337 ± 55 and 376 ± 116 cm-1 for 24h and 48h in the 1D case; and 185 ± 31 and 159 ± 23 cm-1 for 24h and 48h in the 2D case. We ignored the time point of slice #4, which is a clear outlier. The reason is due to difficulties in the experiments, where part of the slice was lost in the subsequent steps, invalidating the calculations (see Figure S 2).

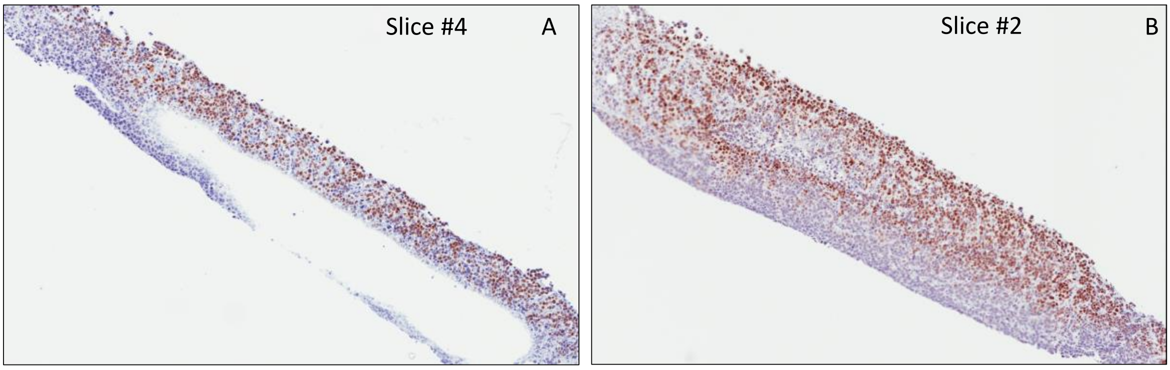

Figure S 2: demonstration of outlier. A) slice #4, a technical outlier. B) slice #2, a normal slice.

Based on these calculations, the fold difference (24h/48h) between the methods were 0.90 and 1.17 for 1D and 2D methods. Via one method k'_R is found to increase, whereas it decreases with the other method. The theoretical framework states that the longer the tissue has been cultured for, the larger the total apparent oxygen uptake rate. In other words, the extension of well oxygenated tissue decreases with the age of the tissue culture. The 2D algorithm recognises more irregularities of the images, being the more reliable method. However, the values obtained for the slices at the different ages show trends opposite to that the theoretical framework and any further comments may be accused of over-interpretation.

Table S 3: results for method (a) 1D HIF1α front comparison and (b) 2D best HIF1α density match comparison. The value * was a technical outliar and was left out of the calculations

		k_R' 1D	k_R' 2D
	h	cm^{-1}	cm^{-1}
Slice #1	24	342	161
Slice #2	24	369	184
Slice #3	24	265	222
Slice #4	24	*13395	222
Slice #5	24	306	173
Slice #6	24	406	150
Slice #7	48	302	141
Slice #8	48	300	149
Slice #9	48	486	153
Slice #10	48	283	124
Slice #11	48	350	176
Slice #12	48	321	188
Slice #13	48	588	181

S4 COMMENTS ON THE GENERALISATION OF THE MULTI-SOURCE OXYGEN DISTRIBUTION

The equation (5) of the main paper was the chosen convenient approach for the calculations in our model. Here, we compare two other alternatives to equation (5) to compute the distance fields by means of the linear average, and thus

$$|x - x_s|_{eq} = \frac{1}{S} \cdot \sum^{S}(|x - x_s|_s), \tag{S6}$$

or the geometric averages, thus

$$|x - x_s|_{eq} = (\prod^{S}|x - x_s|_s)^{1/S}. \tag{S7}$$

To test the validity of these methods we posed a toy example in a 20x20 lattice with 6 elements of stroma in them (Figure S3 A). We calculated the solution of the oxygen maps with a numeric Gauss-Seidel algorithms as well as the analytic approximation with equation (5), fitting the parameter f_{corr} for the maps shown in Figure S3C-D to have minimal discrepancy between them. Further, computing times were recorded at different grid sizes (see Figure S3B), showing the significant advantages of using analytic approximations when computational expensiveness is important (such as in stochastic systems).

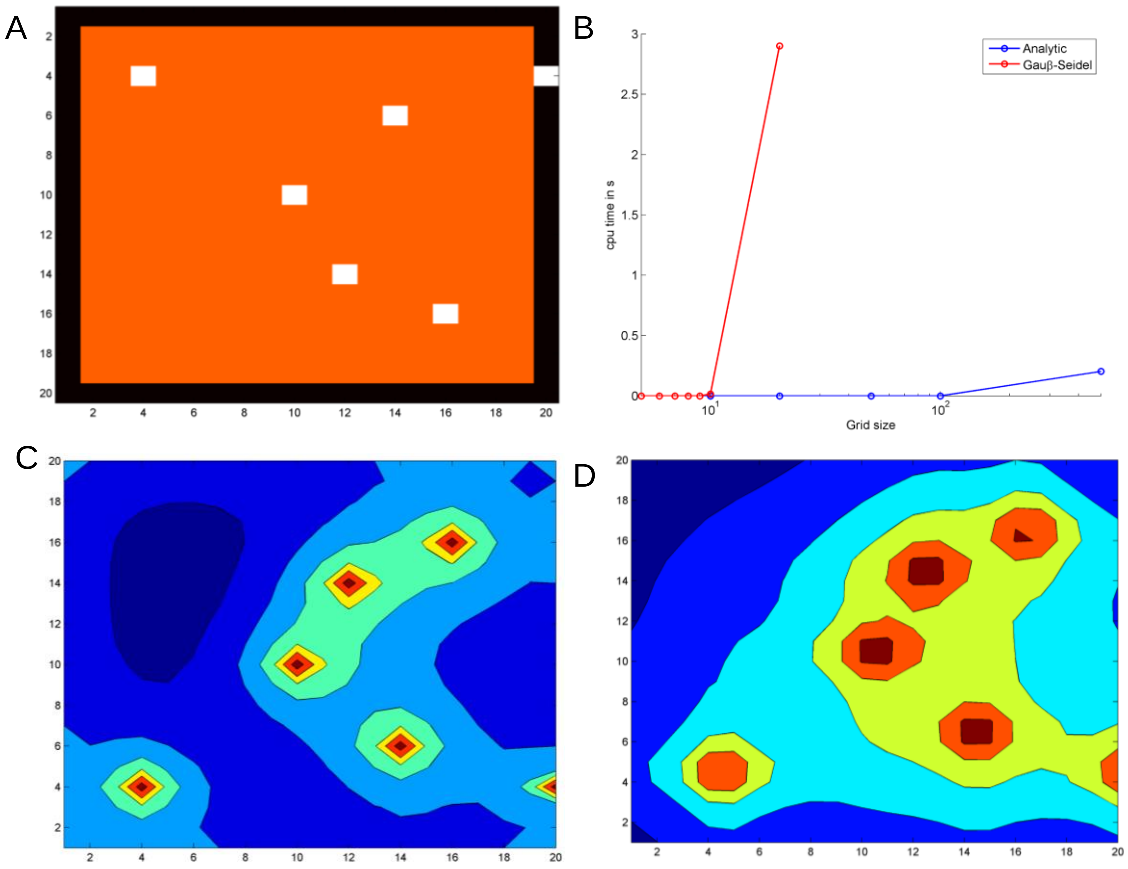

Figure S 3: approximation to the oxygen map. A) map of cells showing their location. White squares are stromal cells, black region is the boundary and the orange region is tumour mass. B) efficiency of the algorithm. CPU time (top) and memory (bottom). C) solution map for the approximation. D) solution map for the discrete Poisson grid solution using a Gauss-Seidel iterative algorithm.

Now evaluating the alternative analytic solutions, both equations (S5) and (S6) show results incongruent with the numeric solution (compare Figure S3D with Figure S4 A, B, D & E) as opposed to the acceptable results produced with equation (5) and represented in Figure S4 C&F. Further, the linear mean would violate the rules of the boundary conditions, which would be

maintained by the geometric mean.

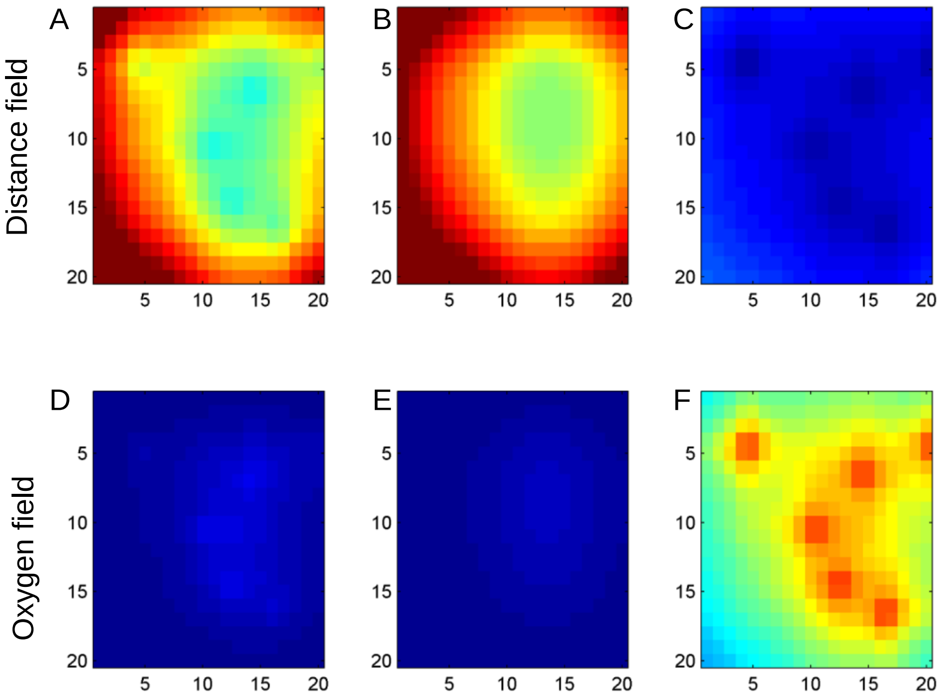

Figure S 4: convolution of distant fields and their oxygen maps. A) geometric mean of distance. B) linear mean of distance. C) inverse sum of distance. D) oxygen map for geometric mean. E) oxygen map for linear mean. F) oxygen map for inverse sum of distance.

S5 Histological quantification of Calu3 and Calu6 models

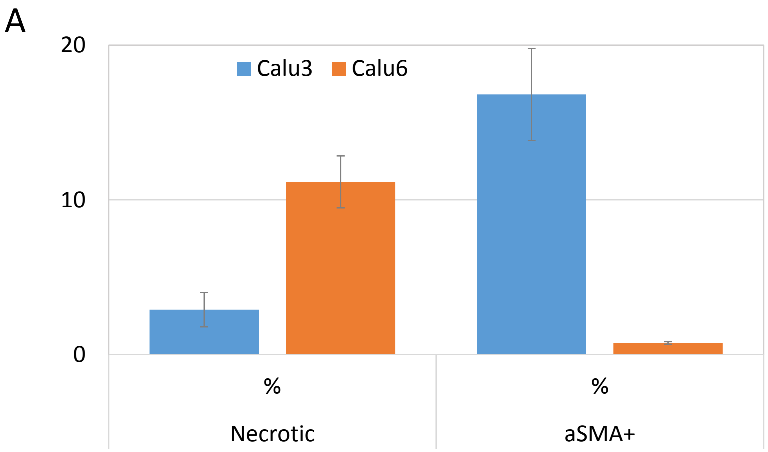

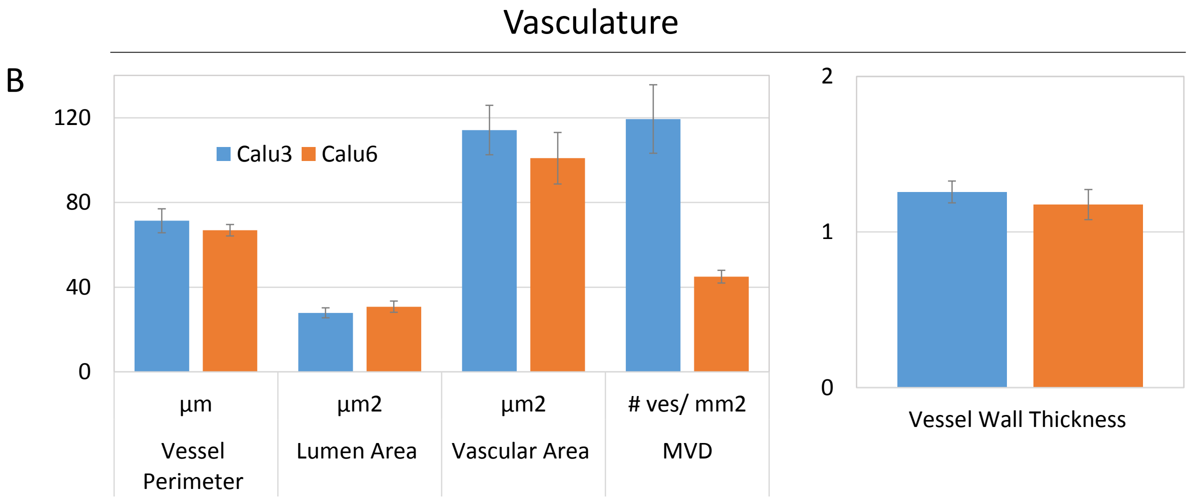

Figure S 5: IHC total quantification for Calu3 (n=4) and Calu6 (n=4). A) necrosis and αSMA+. B) vascular parameters. Error bars show standard deviation.

S6 EXPERIMENTS

All studies were conducted in accordance with UK Home Office legislation, the Animal Scientific Procedures Act 1986 (ASPA) and with AstraZeneca Global Bioethics policy. The analysis in this paper is retrospective, utilising control/untreated animals of different oncology projects within AstraZeneca between 2004 and 2015. All tumour volumes, animal weights and welfare were maintained within the margins fixed by UK and European regulations. No data was generated specifically for this paper

In-vitro experiments

In-vitro experiments were carried out by growing MCF7 cells in 96-well flat bottom plate with 1000 cells in 100µl such that by day 1 the cell confluence is approximately 10 - 20%. Cell growth has been monitored by recording phase images using the IncuCyte ZOOM (EssenBioscience, Germany) live cell imaging system and confluence algorithm. The wells have been imaged every 2 hours to test for percentage of confluence.

Tissue cultures

Tumours were mounted onto the magnetic specimen holder of a Leica VT1200S vibrating blade microtome using cyanoacrylate adhesive. Tumours of small volume were first embedded in low temperature gelling agarose (Thermo) prior to mounting on the specimen holder. Tumour tissue slices were prepared at a thickness of 250 μm.

MCF-7 tumour slices were cultivated in DMEM medium supplemented with glutamine (2 mM; Gibco), penicillin (100 U/ml; Gibco), streptomycin (100 μg /ml; Gibco) and 10% foetal bovine serum (FBS; Gibco). Cultivation was performed at 37°C and 5% CO2 in a humidified atmosphere under low oxygen (1–5% oxygen) or atmospheric oxygen (21% oxygen) conditions. Tissue slices were maintained on Millicell Cell Culture inserts (Merck Millipore, PTFE, pore size 0.4 μm). Half of the medium was replenished after 24 hours. More detail on these methods can be found in Ref [5].

Animal experiments

We used data from the three xenografted cell lines implanted in the SCID and nude mice of both sexes. Briefly, 10^6 to 10^7 human cancer cells, with or without Matrigel (50%), were implanted subcutaneously on the mouse flank. Tumour volumes were calculated from bisecting calliper measurements using the prolate spheroid approximation formula [6]. Tumours were measured

1–3 times weekly. The studies were concluded after two to four weeks. At the end of the study the animals was euthanised and the tumours were excised and were fixed in 10% neutral buffered formalin. Subsequently, the tumours were embedded in paraffin and stored or sent to pathology for staining.

Histopathological staining

IHC was carried out on 5µm tissue sections. The tumour sections were then stained for CD31, αSMA, or HIF1α, counter-stained with Carazzi's hematoxylin, and subsequently scanned. In some cases we used Haematoxyllin & Eosin staining to visualise tissue morphology. Finally, the digital images were quantified with the software ImageScope, Aperio, Leica GmbH, Wetzlar, Germany. We utilised the Aperio packages Microarrays, Genie and ColorDeconvolution algorithms for quantification of the images.

S7 References

1. Ruifrok AC, Johnston DA: **Quantification of histochemical staining by color deconvolution**. *Analytical and quantitative cytology and histology/the International Academy of Cytology [and] American Society of Cytology* 2001, **23**(4):291-299.
2. Henry W: **Experiments on the quantity of gases absorbed by water, at different temperatures, and under different pressures**. *Philosophical Transactions of the Royal Society of London* 1803:29-276.
3. Green DW: **Perry's chemical engineers' handbook**, vol. 796: McGraw-hill New York; 2008.
4. Delgado San Martin JA: **Mathematical models for preclinical heterogeneous cancers**. University of Aberdeen; 2016.
5. Davies EJ, Dong M, Gutekunst M, Närhi K, van Zoggel HJ, Blom S, Nagaraj A, Metsalu T, Oswald E, Erkens-Schulze S: **Capturing complex tumour biology in vitro: histological and molecular characterisation of precision cut slices**. *Scientific reports* 2015, **5**.
6. Delgado San Martin J, Worthington P, Yates J: **Non-invasive 3D time-of-flight imaging technique for tumour volume assessment in subcutaneous models**. *Laboratory animals* 2014:0023677214562653.
